# [3.3]Dithia-bridged cyclophanes featuring a thienothiophene ring: synthesis, structures and conformational analysis

**DOI:** 10.3762/bjoc.5.74

**Published:** 2009-12-08

**Authors:** Sabir H Mashraqui, Yogesh Sanghvikar, Shailesh Ghadhigaonkar, Sukeerthi Kumar, Auke Meetsma, Elise Trân Huu Dâu

**Affiliations:** 1 Department of Chemistry, University of Mumbai, Vidyanagari, Santacruz E, Mumbai 400 098; 2 Department of Chemistry, State University of Groningen, Nijenborg 9747 AG, Groningen, The Netherlands; 3 Institut de Chimie des Substances Naturelles, C.N.R.S., 91190 Gif-sur-Yvette, France

**Keywords:** conformational energy minimization, [3.3]dithia-bridged cyclophanes, dynamic NMR analysis, thieno[2,3-*b*]thiophene, X-ray crystal structure

## Abstract

The synthesis of [3.3]dithia-bridged cyclophanes **7**, **9** and **11** incorporating a fused heterocycle, thieno[2,3-*b*]thiophene is described. The structures are established by ^1^H NMR analysis and, in the case of **11**, also by single crystal X-ray crystallography. Conformational analysis by variable temperature NMR suggests that cyclophanes **7**, **9** and **11** exhibit conformationally rigid bridges and rings at least up to 130 °C. Energy minimization of **11** revealed ***anti*-11** to be the most stable conformation. Although, the computed energy difference between the most stable conformation ***anti*-11** and the next higher energy conformation ***syn-anti*-11** is only 2.98 kJ/mol, it is intriguing that **11** does not exhibit thia-bridge inversion even at elevated temperatures.

## Introduction

The synthesis, molecular structures and conformational dynamics of short-bridged cyclophanes continue to engage interest in supramolecular chemistry [1–4]. The study of the conformational dynamics of cyclophanes has greatly enriched our fundamental understanding with regard to the structural features governing the energy barriers to ring inversion processes in these molecules [5–6]. Structural factors that have bearing on the conformational energy barriers of the ring and bridges in cyclophanes include the type the rings, nature of inner substituents and the length of the bridges encompassing the ring(s). Temperature dependent conformational behaviors of a diverse variety of [1.3]dithia-cyclophanes have been studied extensively and depending upon structural features both conformationally restricted and freely interconverting thia-bridged systems have been identified [7–17].

**Figure 1:**
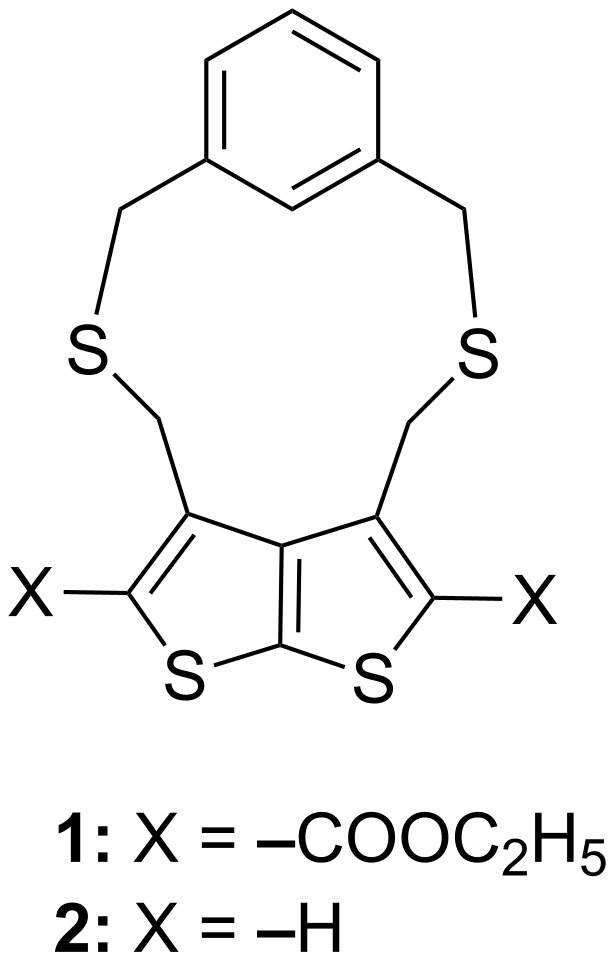
Conformationally flexible 3, 4-bridged dithia-thienothiophenophanes.

Cyclophanes incorporating simple heteronuclei such as furan, thiophene, pyridine, pyridazine and benzothiazole are abundantly known [1–3]. Despite the known structural diversity and complexity of cyclophanes, it is surprising that cyclophanes featuring fused heteronuclei have scarcely been studied [18–20]. Lately, we have been exploiting the thieno[2,3-*b*]thiophene ring, a fused heterocycle towards creating potentially interesting intramolecular charge transfer [21], nonlinear optics [22] and axially chiral systems [23]. We have also recently incorporated a thienothiophene ring within the cyclophane framework via its 3,4-positions, leading to the synthesis of *meta* type 1,3-dithia-bridged thienothiophenophanes **1**–**2** [24]. Cyclophanes **1**–**2** (Figure 1) have been found to exhibit thia-bridge inversion down to −50 °C with an estimated energy barrier >25 kJ mol^−1^. In the present work, we deemed it of interest to exploit the other available positions of the thienothiophene ring, namely the 2, 5 positions to access a new set of bridged [3.3]dithia-thienothiophenophanes **7**, **9** and **11**. Our main objective in the synthesis of these cyclophanes was to evaluate their structures and conformational properties.

## Results and Discussion

### Synthesis of dithia-thienothiophenophanes **7**, **9** and **11**

**Scheme 1:**
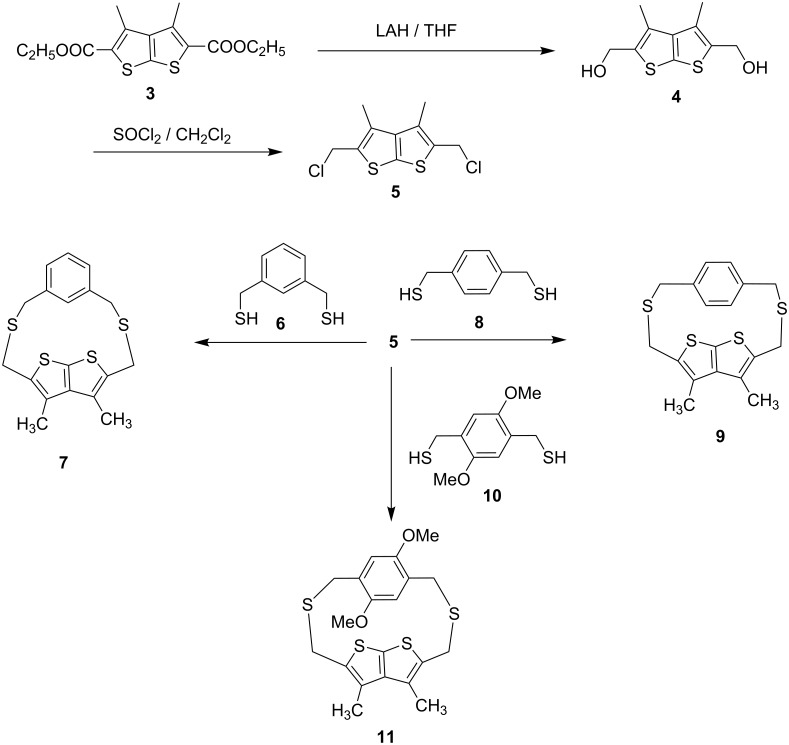
Synthetic scheme for compounds **7**, **9** and **11**.

2,5-Bis(chloromethyl)-3,4-dimethylthieno[2,3-*b*]thiophene (**5**) required as a precursor to construct cyclophanes **7**, **9** and **11** was prepared as outlined in Scheme 1. The starting material, thienothiophene diester **3** was readily prepared in multigram quantities by a modification of our procedure [25]. Reduction of **3** with LiAlH_4_ in dry THF, followed by basic aqueous work-up gave diol **4** in 91% yield. The reaction of **4** with SOCl_2_ in dry dichloromethane (5–10 °C, 5 h), followed by solvent removal and crystallization from petroleum ether afforded dichloride **5** in 88% yield, m.p. 161–163 °C. The coupling reaction of **5** with m-xylenedithiol **6** was carried out in dry DMF/K_2_CO_3_ under high dilution conditions and under N_2_ atmosphere. The crude solid obtained on work-up was purified by column chromatography on SiO_2_ affording the desired *meta* dithia[3.3]cyclophane **7**, m.p. 247–250 °C as a colorless solid in 57% yield. Likewise, the *para* dithia-bridged thienothiophenophanes **9** and **11** were prepared by coupling dichloride **5** with dithiols **8** and **10** respectively under the conditions described for **7**. The crude products from these reactions were purified by column chromatography on SiO_2_ to furnish the corresponding dithia-cyclophanes **9** and **11** in 50% and 10% yields respectively.

### NMR assignments and conformational analysis of thienothiophenophanes **7**, **9** and **11**

The ^1^H NMR (300 MHz) spectrum of dithia-cyclophane **7** revealed two AB spin systems (4H each) in the range of δ 3.21–4.30 due to the geminal couplings of the bridged –CH_2_- protons. A singlet centered at δ 2.31 accounted for the C3/C4 methyl groups. The adjacent aromatic H4, H5 and H6 protons appeared as a multiplet (3H) centered at δ 7.32, whereas the isolated internal H2 proton appeared as a sharp singlet (1H) at δ 6.03. The higher field position of the internal H2 compared to the chemical shifts of H4-H5-H6 protons is presumably due to the shielding anisotropic effect of the facing thienothiophene ring.

The ^1^H NMR (700 MHz) spectrum of dithia-cyclophane **9** showed two sets of AB type spin systems, comprising doublets at δ 3.65 (2H, *J* = 15 Hz), 3.72 (2H, *J* = 14 Hz), 3.73 (2H, *J* = 15 Hz) and 4.08 (2H, *J* = 14 Hz). The appearance of two AB type spin systems implies that the methylene protons attached to one side of the bridge are magnetically identical with their respective counterparts on the other side of the bridge. A singlet located at δ 2.19 is due to the methyl protons, whereas the phenyl ring protons appear as sharp singlets at δ 6.84 and 6.95 (2H each). Appearance of two singlets for the *para* bridged phenyl ring implies that the two sides of the phenyl ring are subject to differing magnetic fields on account of the unsymmetrical nature of the facing thienothiophene ring.

The ^1^H NMR of **11** showed a 16 line resonance (δ 3.36 to 4.30) expected for the AB type coupling of its four unique sets of bridge methylene protons. Each of the −CH_3_ groups and −OCH_3_ groups appear as separate singlets observed at δ 2.20, 2.31 and 3.75, 3.82, respectively. The nonequivalence of otherwise chemically identical pair of −CH_3_ as well as −OCH_3_ group of protons can be attributed to the differing magnetic environments around these groups created by the facing thienothiophene and the phenyl rings. Although, in analogy to the −CH_3_ and −OCH_3_ protons, we expected the phenyl ring protons to also appear as two separate singlets, however in practice we observe only one singlet at δ 6.80 (2H). This may presumably be due to their coincidental chemical shift equivalence.

The bridge −CH_2_− protons in dithia-bridged cyclophanes **7**, **9** and **11** resonate as geminally coupled AB systems. From these results, we infer that the bridge inversions in these molecules are restricted on the NMR time scale at ambient temperatures. Furthermore, since phenyl ring protons in **9** and methoxy protons in **11** resonate at two different chemical shifts, we can conclude that aryl ring rotations in these molecules also seem to be conformationally restricted. Higher temperatures might surpass the conformational energy barrier, allowing the molecules to undergo free bridge and ring inversions [26]. In the event of free bridge rotations, the geminally coupled systems would coalesce into two sharp singlets corresponding to the two different sets of the bridged −CH_2_− protons. On the other hand, ring rotation in **9** and **11** would convert separate signals for the phenyl ring and methoxyl protons into singlets due to the fast interconversion of ring conformers. Accordingly, we recorded variable temperature NMR spectra of dithia-cyclophanes **7**, **9** and **11** at different intervals from room temperature to 130 °C in DMSO-*d*_6_. However, no changes were noticeable in the multiplicities of the bridge −CH_2_− protons with the aryl ring and methoxy protons also retaining their separate chemical shift positions. These observations suggest the absence of bridge flippings and ring rotations in these systems at least up to 130 °C with an estimated conformational energy barrier of >100 kJ mol^−1^ [27]. The presence of substituents on the thienothiophene ring (as well as on the phenyl ring in **11**) and the absence of conformational inversion endow these with molecular chirality rendering them potentially resolvable.

Temperature dependent conformational behaviors have been reported for a number of unsubstituted [1.3] dithia-cyclophanes [5–6]. However, replacing internal hydrogen atoms of the thienothiophene with a bulky substituent viz. −CH_3_ in [1.3]dithia- and related cyclophanes raises the conformational energy barriers sufficiently to render such systems conformationally immobile [28–31]. By analogy, it is reasonable to assume that the conformational restrictions of the bridges and rings observed in the presently synthesized cyclophanes could at least in part be due to the presence of methyl substituents on the thienothiophene rings in **7** and **9** and both methyl and methoxyl substituents for case of **11**.

### Single X-ray Crystal structure and conformational search of **11**

**Figure 2:**
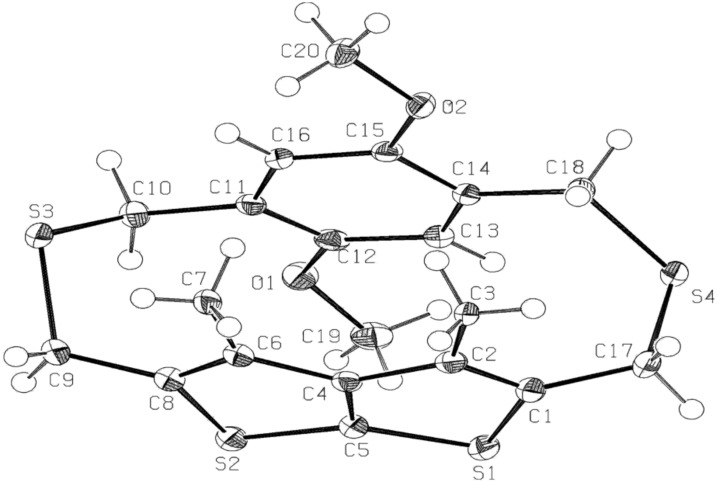
ORTEP plot of the crystal structure of **11**. Important parameters: Bond length (Å) : C8–C9 = 1.492, C1–C7 = 1.497, C10–C11 = 1.517, C14–C18 = 1.522; Bond Angles (deg): C4, C6, C7 = 124.70, C1, C2, C3 = 125.03, C10, C11, C12 = 120.31, C13, C14, C18 = 123.14, C10, S3, C9 = 104.13; C18, S4, C17 = 101.14, C16, C11, C10 = 121.12 C15, C14, C18 = 118.43; Dihedral Angles (deg): C18, C14, C15, C16 = −174.40(13), C10, C11, C12, C13 = −175.43(14), C10, C11, C16, C15 = 178.80(13), C15, C16, C11, C12 = −2.7(2), C12, C13, C14, C15 = −3.1(2), C8, C6, C4, C6 = 168.50(16), C1, C2, C4, C6 = −167.65(16), C5, C4, C2, C1 = 5.29.

The single-crystal structure of **11** was deduced by X-ray crystallography [32]. The space group of the molecular structure is P2_1_/n, meaning the point group is 2/m. An ORTEP drawing is shown in Figure 2 and the relevant crystallographic parameters are compiled in Table 1. The torsion angle C18–C14–C15–C16 and C10–C11–C12–C13 are −174.40(13)° and −175.80(13)° indicating that the benzene ring is slightly inwardly bend. The dihedral angles C8–C6–C4–C5 and C5–C4–C2–C1 are −2.00(19)° and 2.85(19)°, respectively; thereby implying that thienothiophene ring is also slightly inwardly bent. The thia bridges are anti oriented with respect to one another, and so are the two methoxyl groups. The angle of 104.13° for C10–S3–C9 is slightly larger than 101.13° for C18–S4–C17, presumably to minimized steric interaction between the methoxyl and methyl substituents on either sides of the bridges. The transannular distances between the bridgehead carbons C11–C6 and C14–C2 are 3.37 Å and 3.32 Å, respectively and the phenyl and thienothiophene rings are stacked with the former slightly displaced from the center of the latter.

**Table 1:** Summary of crystallographic data and refinement details.

Empirical formula	C_20_H_22_O_2_S_4_
Formula mass	422.66
Crystal color and habit	colorless, block
Crystal size [nm]	0.23 × 0.21 × 0.19
Crystal system	monoclinic
a [Å]	8.2943(5)
b [Å]	13.8204(9)
c [Å]	16.679(1)
β [°]	97.690(1)
V [Å^3^]	1894.7(2)
ρ_calcd._ [g/cm^3^]	1.482
F(000)	888
μ [cm^−1^]	5.14 (Mo-Kα)
2θ_max_ [°]	55.0 (I > 3.0σ)
No. of reflections	4039

In order to evaluate the energy minimized conformations for **11**, we performed the conformational analysis by random search Monte Carlo method [33] and optimized by PRCG Conjugated Gradient molecular mechanics minimization using the Macromodel version 5.5 program [34], MM2 force field [35] and GB/SA chloroform solvation [36]. Two thousand conformations were generated from which we have selected three of the most stable conformations within 12.55 kJ/mol from the global minimum.

**Scheme 2:**
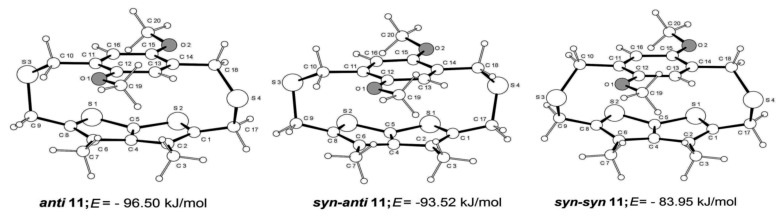
Computed three most stable conformations of **11** and their calculated energies.

As depicted in Scheme 2, the notable difference between these conformations is the relative orientation of the bridging sulfur atoms with respect to the thienothiophene ring. The conformation designated as ***anti*-11** is unsymmetrical with the bridging sulfur atoms being anti-oriented i.e. facing away from each other. The conformations ***syn-anti*-11** and ***syn-syn*-11** both have their bridge ‘S’ atoms symmetric about a plane bisecting the two rings, though they are not symmetric to the plane. Both conformations, ***syn-anti*-11** and ***syn-syn*-11** possess *syn*-oriented sulfur bridges, however: for the former the bridging sulfur atoms are pointing away from the sulfur atoms of the thienothiophene ring, while for the latter, the bridging sulfur atoms and the thienothiophene ring sulfur atoms are facing the same side. The key dihedral angles of these conformations together with those of **11** derived from the X-ray data are compiled in Table 2.

**Table 2:** Selected computed dihedral angles (deg) of ***anti*-11**, ***syn-anti*-11**, and ***syn-syn*-11** and those of **11** derived from the X-ray crystallography.

	***anti*-11**	***syn-anti*-11**	***syn-syn*-11**	**11**

*E* in kJ/mol	−96.50	−93.52	−83.95	–
Δ*E* in kJ/mol	0.00	2.98	12.55	–
S2–C5–S1–C1	−162.7°	+161.0°	−162.9°	168.61° (13)
C5–S1–C1–C17	+169.8°	−168.5°	+170.3°	−165.12° (12)
S1–C1–C17–S4	−61.3°	+51.8°	−120.4°	73.25° (13)
C1–C17–S4–C18	−67.0°	+71.9°	+63.0°	56.29° (12)
C17–S4–C18–C14	+78.9°	−69.8°	−74.9°	−85.25° (12)
C11–C10–S3–C9	+82.9°	+80.2°	+82.5°	−78.54° (13)
C10–S3–C9–C8	−58.2°	−68.3°	−60.3°	49.78° (13)
S3–C9–C8–S2	+113.3°	−61.8°	+112.5°	−106.16° (12)
C9–C8–S2–C5	−169.8°	+169.2°	−168.5°	169.94° (13)
C8–S2–C5–S1	+163.6	−161.7	+162.4	−169.57 (13)

The conformation ***anti*-11** is the lowest energy structure with ca. E of −96.50 kJ/mol, while ***syn-anti*-11** and ***syn-syn*-11** are of relatively higher energy by ca. 2.98 and 12.55 kJ/mol, respectively. Inspection of Table 2 reveals that while some of the computed dihedral angles of the three most stable conformations are quite close to each other, the computed dihedral angles viz. S1–C1–C17–S4, C17–S4–C18–C14, C10–S3–C9–C8 and C8–S2–C5–S1 for the most stable ***anti*-11** are broadly similar to those of the cyclophane **11** derived from the X-ray data. Consequently, there is good correspondence between the X-ray crystal structure and the most stable conformation ***anti*-11** computed from the conformation energy minimization. Although, the Δ*E* between the most stable conformation ***anti*-11** and the next higher energy conformation ***syn-anti*-11** is only 2.98 kJ/mol, it is intriguing that **11** does not exhibit thia-bridge inversion even at elevated temperatures. The flipping of the thia-bridges in **11** as well as other cyclophanes described in the preceding section could be inhibited if the transition state structures have free energies of activation >100 kJ/mol [5]. This aspect is currently being examined using molecular modeling approach.

## Conclusion

In conclusion, we have reported the synthesis and structures of [3.3]dithia-bridged thienothiophenophanes **7**, **9**, and **11**. Variable temperature ^1^H NMR analysis revealed the absence of bridge flipping up to 130°C in these molecules. Phenyl ring rotation in cyclophanes **9** and **11** is also ruled out since separate singlets associated with phenyl protons (in case of **11**, also the –OCH_3_) failed to coalesce up to 130 °C. The structure of **11** was also deduced by a single crystal X-ray crystallography. The most stable conformation *anti***-11** derived from conformational minimization possessed broad structural similarity with that of the X-ray crystal structure.

## Experimental

The chemicals and spectroscopic grade solvents were purchased from S.D.F. Chemicals (India) and Aldrich and used as received. IR spectra were recorded on a Shimadzu FTIR-420 spectrophotometer. ^1^H NMR spectra were scanned in CDCl_3_ using a Bruker 300 or 700 MHz spectrometer with TMS as an internal standard. Elemental analyses were done on a Carlo Erba instrument EA-1108 Elemental analyzer.

### Preparation of 3,4-dimethyl-2,5-bis(hydroxymethyl)thieno[2,3-*b*]thiophene (**4**)

A solution of diester thienothiophene **3** (1.56 g, 5 mmol) dissolved in dry THF (20 ml) was added to a slurry of LiAlH_4_ (950 mg, 25 mmol) prepared in dry THF (50 ml) at room temperature over a period of 1 h. After stirring for 24 h at room temperature, the reaction mixture was cooled in an ice-salt bath and hydrolyzed by successive additions of water (1 ml), 15% sodium hydroxide (1 ml) and water (2.5 ml) with vigorous stirring. The reaction mixture was then filtered through celite and the filtered cake washed thoroughly with dry THF. The filtrate was concentrated to give diol **4** as a colorless solid (1.03 g, 91% yield); mp: 156–158 °C; IR (KBr) cm^−1^: 3250, 2921, 2861, 1525, 1443, 1418, 1399, 1353, 1234, 1191, 1125, 1113, 1003, 949, 700. ^1^H NMR (DMSO, 300MHz) δ : 5.20(s, 2H, –OH), 4.60(s, 4H, −CH_2_−), 2.40(s, 6H, −CH_3_). Anal. Calcd for C_10_H_12_O_2_S_2_: C, 52.63; H, 5.26; S, 28.07. Found C, 52.78; H, 5.49; S, 28.23.

### Preparation of 3,4-dimethyl-2,5-bis(chloromethyl)thieno[2,3-*b*]thiophene (**5**)

Diol **4** (1.14 g, 5 mmol) was stirred in dry dichloromethane and freshly distilled thionyl chloride (1.0 ml, 13.6 mmol) was added dropwise at 5–10 °C. The reaction mixture was allowed to warm to room temperature over 5 h. The reaction mixture was then concentrated and the obtained brown solid was boiled with light petroleum ether. The supernatant was transferred to a dry flask and the clear solution refrigerated overnight to furnish dichloride **5** as white–brownish solid (1.15 g, 88% yield); mp 161–163 °C; IR (KBr) cm^−1^: 2923, 1520, 1436, 1422, 1395, 1261, 1174, 1131, 1112, 862, 811, 673. ^1^H NMR (CDCl_3_, 300 MHz) δ: 4.70 (s, 4 H, –CH_2_), 2.50 (s, 6H, –CH_3_). Anal. Calcd for C_10_H_10_Cl_2_S_2_: C, 45.28; H, 37.74, S, 24.15, Cl, 26.42 Found C, 45.40; H, 37.57; S, 24.28, Cl, 26.35.

### Preparation of dithia[3]metacyclo[3](2,5)thienothiophenophane **7**

A solution of dichloride **5** (265 mg, 1 mmol) and 1,3-bis(mercaptomethyl)benzene (**6**) (170 mg, 1 mmol) in dry DMF (50 ml) was added dropwise to a stirred mixture of dry DMF (100 ml) containing anhyd. K_2_CO_3_ (250 mg) at 60–70 °C over 8 h under N_2_ atmosphere. The reaction mixture was further stirred at this temperature for 16 h. It was then allowed to cool and filtered through a pad of celite. The filtrate was concentrated in vacuum to give a dark–brown colored residue. The residual solid was extracted in chloroform, washed with water and dried over anhyd. Na_2_SO_4_. The crude product obtained on concentration was purified by column chromatography on SiO_2_ using petroleum ether–chloroform (2:1) as an eluant. The cyclophane **7** was obtained as a colorless solid, (207 mg, 57%), m.p. 247–250 °C; IR (KBr) cm^−1^: 3008, 2947, 2907, 1583, 1482, 1428, 1396, 1228, 1215, 1082, 1009, 978, 780, 711. ^1^H NMR (CDCl_3_, 300 MHz) δ : 7.02 (s, 3H, Ph–H), 6.03 (s, 1H, Ph–H), 4.30 (d, 2H, *J* = 16Hz, –CH_2_-thienothiophene), 3.82 (d, 2H, *J* = 16Hz, −CH_2_-thienothiophene), 3.56 (d, 2H, *J* = 16 Hz, −CH_2_-phenyl), 3.21 (d, 2H, *J* = 16Hz, −CH_2_-phenyl), 2.31(s, 6H, −CH_3_). Mass : m/e 362. Anal. Calcd for C_18_H_18_S_4_: C, 59.67; H, 4.97; S, 35.36. Found C, 59.51; H, 4.93; S, 35.61.

### Preparation of dithia[3]paracyclo[3](2,5)thienothiophenophane **9**

Dichloride **5** (265 mg, 1 mmol) and 1,4-bis-(mercaptomethyl)benzene (**8**) (170 mg, 1 mmol) were coupled as described for **7**. The crude product was chromatographed on SiO_2_ column using petroleum ether–chloroform (2:1) as an eluant to afford cyclophane **9** as a colorless solid (180 mg, 50%); mp 210–212 °C; IR (KBr) cm^−1^ : 2922, 1525, 1504, 1445, 1414, 1397, 1376, 1215, 1122, 1101, 1006, 892, 838, 791, 776, 722. ^1^H NMR (700 MHz, CDCl_3_) δ: 6.95 (s, 2H, Ph–H), 6.84 (s, 2H, Ph–H), 4.08 (2H, *J* = 14 Hz), 3.73 (2H, *J* = 15 Hz), 3.72 (2H, *J* = 14 Hz), 3.65 (2H, *J* = 15 Hz), 2.19 (s, 6H, −CH_3_). ^13^C NMR (300 MHz, CDCl_3_) δ: 147.37, 136.20, 135.11, 133.80, 129.08, 126.07, 125.22, 36.07, 30.87, 13.72. Mass: m/e 362. Anal. Calcd for C_18_H_18_S_4_: C, 59.67; H, 4.97; S, 35.36. Found C, 59.73; H, 4.81; S, 35.49.

### Preparation of dimethoxydithia[3]paracyclo[3](2,5)thienothiophenophane **11**

Dichloride **5** (265 mg, 1 mmol) and 2,5-dimethoxy-1,4-bis(mercaptomethyl)benzene (**10**) (156 mg, 1 mmol) were coupled as described for **7**. The crude product was chromatographed on SiO_2_ using petroleum ether–chloroform (2:1) as an eluant to afford cyclophane **11** as a colorless solid (23 mg, 10% yield); mp 252–253 °C. IR (KBr) cm^−1^: 2988, 2950, 2836, 1606, 1576, 1492, 1459, 1446, 1409, 1308, 1231, 1196, 1042, 1007, 890, 864, 769, 749. ^1^H NMR (300 MHz, CDCl_3_) δ : 6.80 (s, 2H, Ph–H), 4.30 (d, 1H, *J* = 14 Hz, –CH_2_), 4.14 (d, 1H, *J* = 17 Hz, –CH_2_), 4.02 (d, 1H, *J* = 18 Hz, –CH_2_), 3.9 (d, 1H, *J* = 17 Hz, –CH_2_), 3.81 (d, 1H, *J* = 14 Hz, –CH_2_), 3.58 (d, 1H, *J* = 15 Hz, –CH_2_), 3.44 (d, 1H, *J* = 18 Hz, –CH_2_), 3.36 (d, 1H, *J* = 14 Hz, –CH_2_), 3.75 (s, 3H, –OCH_3_), 3.80 (s, 3H, –OCH_3_), 2.22 (s, 3H, –CH_3_), 2.29 (s, 3H, –CH_3_). Mass: m/e 422. Anal. Calcd for C_20_H_22_O_2_S_4_: C, 56.87; H, 5.21; S, 30.33. Found C, 56.94; H, 5.45; S, 30.19.
